# Is mechanical loading essential for exercise to preserve the aging immune system?

**DOI:** 10.1186/s12979-021-00238-9

**Published:** 2021-06-05

**Authors:** Richard J. Simpson, Graham Pawelec

**Affiliations:** 1 Department of Nutritional Sciences, The University of Arizona, Tucson, USA; 2 Department of Pediatrics, The University of Arizona, Tucson, USA; 3 Department of Immunobiology, The University of Arizona, Tucson, USA; 4 The University of Arizona Cancer Center, Tucson, Arizona USA; 5 Department of Immunology, University of Tübingen, Tübingen, Germany; 6 Health Sciences North Research Institute, Sudbury, Ontario Canada

Exercise improves all aspects of health, including immunity. It is well known that exercise at a level consistent with most national physical activity guidelines can improve immune responses to vaccination, lower the incidence of infection and latent viral reactivation, lower chronic low-grade inflammation, and is associated with a reduction in many hallmark features of an aging immune system, especially in older adults [1]*.* The lymphoid compartment is particularly susceptible to age-related changes and also appears to be most responsive to exercise. For instance, the blood T-cell compartment in physically active compared to sedentary older adults express fewer markers of differentiation and exhaustion and have higher numbers and proportions of naïve T-cells. Moreover, T-cells (particularly CD8+ T-cells) from those who are physically active have an enhanced ability to secrete IL-2 and proliferate in response to antigenic stimulation, while NK-cells in those who are physically active have an increased ability to secrete effector cytokines and perform anti-tumor cytotoxic functions [1]*.* While the beneficial effects of exercise on immunity are apparent, details of the mechanisms responsible have remained opaque. We do know that exercise evokes the release of several cytokines from contracting skeletal muscle (i.e. ‘myokines’) that can have a direct impact on the peripheral lymphoid compartment. For instance, IL-7 is released from muscle during exercise which can, in turn, help preserve thymic mass and T-cell production to potentially help sustain the naïve T-cell compartment with advancing age. Similarly, IL-15 released by muscle can maintain peripheral T-cell and NK-cell numbers and enhance NK-cell cytotoxicity and cytokine secretion. While these links between exercise-released myokines and immune maintenance are intuitive and have been demonstrated in cross-sectional studies, they have yet to be tested mechanistically [1]*.* Furthermore, the optimal exercise prescription/dose as a function of the FITT (frequency, intensity, time and type) criteria and principles of exercise training (e.g. overload, progression) required to evoke positive changes in immunity remain elusive.

A recent study in mice by Shen et al. indicates that the positive effects of exercise on immunity could be due to an enhanced production of common lymphoid progenitors (CLPs) facilitated by mechanical loading [2]*.* The authors showed that bone marrow stromal cells expressing the leptin receptor and osteolectin (an osteogenic growth factor) also express PIEZO1, a mechanoreceptor triggering stem cell factor production by osteogenic progenitors, which in turn helps maintain CLPs. Deleting PIEZO1 from osteolectin+ cells depleted osteolectin+ cells and CLPs. The authors further showed that deleting stem cell factor (SCF) - a critical growth factor for the maintenance of hematopoietic stem cells and early restricted progenitors - from osteolectin+ cells did not affect hematopoietic stem cells or most restricted progenitors, but did deplete CLPs and impaired lymphopoiesis, bacterial clearance and survival following acute bacterial infection. Leptin receptor+ osteolectin+ cells are osteogenic progenitors that are short lived and divide rapidly, decreasing in number with age and increasing in number after fracture. With this in mind, Shen et al. found that voluntary wheel running increased the frequencies of peri-arteriolar osteolectin+ cells and CLPs, whereas hindlimb suspension (a common rodent model of mechanical unloading) decreased the numbers of these cells. Collectively, their results show that a peri-arteriolar bone marrow niche for osteogenesis and lymphopoieses is depleted during aging, essential for resolving bacterial infections, and maintained by mechanical loading provided by exercise.

Shen et al. have shed light on a potential mechanism by which exercise can preserve immunity during aging. A longstanding hypothesis in the exercise immunology literature is that the peripheral naïve T-cell compartment is better preserved in those who are physically active due to exercise evoking selective apoptosis of terminally differentiated/exhausted T-cells, leaving ‘space’ for the production of new recruits by hematopoiesis and thymopoeisis [3]*.* Indeed, the adoptive transfer of exercise-induced apoptotic lymphocytes in mice has been shown to evoke a bone marrow response to produce new progenitor cells, and the findings reported by Shen et al. that running exercise in mice increases the production of CLPs provides further support for this hypothesis. Moreover, hematopoiesis is skewed away from the production of lymphoid cells and towards myeloid cells in older animals and humans, with potential clinical consequences [4]*.* Whether or not exercise training can mitigate the effects of aging on myeloid skewing could have important implications for immune health in older adults and should be experimentally tested. The findings by Shen et al. that mechanical loading and unloading evoked divergent effects on the frequencies of osteolectin+ cells and CLPs has opened a new line of mechanistic enquiry. If mechanical load is the major ingredient by which exercise promotes lymphopoieses then this would raise a number of questions regarding the type of exercise that should be performed to best optimize immunity. For instance, perhaps high load bearing exercises such as running and weight training could be a better stimulus to promote lymphopoieses and prevent myeloid skewing than low load bearing exercises like cycling or swimming. This question could be addressed through randomized clinical trials in older adults involving different modes of exercise training. As a human model of mechanical unloading, the effects of exercise training on hematopoiesis in microgravity could be determined in astronauts onboard the International Space Station (ISS). Space travel is known to evoke immune dysregulation and lead to the frequent reactivation of latent herpesviruses including CMV and EBV, as well as VZV. It was shown recently that physical fitness (both cardiorespiratory and muscular endurance) protected ISS crewmembers from latent viral reactivation, particularly if the astronauts engaged in enough exercise in orbit to prevent physical deconditioning on return to Earth [5]*.*

Finally, there is a need to determine how the production of CLPs with exercise influences the overall function of the peripheral lymphoid compartment. Certainly, increased CLP production could help prevent the aforementioned myeloid skewing and maintain the frequency and diversity of the naïve T-cell compartment, but a key benefit of exercise on the immune response is the frequent mobilization and redistribution of effector lymphocytes that occurs with every bout. NK-cells and CD8+ T-cells mobilized with exercise have been shown to augment immune surveillance and these mobilized cells have been shown to infiltrate tumors to inhibit tumor growth in several murine cancer models [6]*.* This response is critically dependent on the actions of catecholamines binding to beta-2 adrenergic receptors on the effector lymphocytes. Lymphocyte adrenergic receptor density and sensitivity declines with age, perhaps explaining why older adults do not redeploy as many effector lymphocytes with exercise compared to their younger counterparts. This may mean older adults having less of an exercise benefit with regards to immune surveillance due to adrenergic receptor desensitization. Whether or not replenishment of the peripheral T-cell compartment by CLPs due to load bearing exercise will result in more effector lymphocytes that are capable of responding to catecholamines is not known. These novel findings by Shen et al. may help us unravel new mechanisms by which exercise appears to preserve the aging immune system.

## Data Availability

Not applicable.
